# Treatment outcome of anti-angiogenesis through *VEGF*-pathway in the management of gastric cancer: a systematic review of phase II and III clinical trials

**DOI:** 10.1186/s13104-018-3137-8

**Published:** 2018-01-12

**Authors:** Brian Mawalla, Xianglin Yuan, Xiaoxiao Luo, Phillip L. Chalya

**Affiliations:** 1 Department of Oncology, Huazhong University of Science and Technology, Tongji Medical College, Tongji Hospital, Wuhan, Hubei China; 2 Department of Surgery, Bugando Medical Centre, Mwanza, Tanzania

**Keywords:** Gastric cancer, Anti-angiogenesis, Management, Treatment outcome

## Abstract

**Objectives:**

Advanced gastric cancer poses a therapeutic challenge worldwide. In randomised clinical trials, anti-VEGF has been reported as an essential agent for the treatment of advanced gastric cancer. This review aims at assessing the treatment outcome of anti-angiogenesis therapy through the VEGF pathway in the management of patients with advanced gastric cancer.

**Results:**

During this review, 38 clinical trials were identified. Of these, 30 clinical trials were excluded, leaving eight trials of phase II and III. Ramucirumab, as a second line treatment of advanced gastric cancer, decreases the risk of disease progression (37–52%) and death (19–22%). Compare ramucirumab and bevacizumab in combination with traditional chemotherapy; ramucirumab has shown to improve progression-free survival and overall survival. Apatinib tyrosine kinase inhibitor combined with traditional chemotherapy has shown to improve overall response rate and progression-free survival with marginal improvements in overall survival. Chemotherapy, in combination with anti-VEGF drugs, in the management of advanced gastric cancer significantly improves the outcome of overall response rate, progression-free survival and overall survival when compared to chemotherapy alone. Therefore, we recommend that anti-VEGF drugs are the drugs of choice in the management of patients with advanced gastric cancer.

## Introduction

Gastric cancer represents a significant cancer burden worldwide and is associated with poor prognosis [1]. Globally cancer statistics reported that gastric cancer is the fifth most common cancer and the third most common cancer-related deaths [2]. Surgical resection followed by adjuvant chemotherapy with or without radiotherapy is of paramount importance to achieve cure in patients presents with the early stage of gastric cancer [3]. However, almost two-thirds of patients with gastric cancer diagnosed when the disease is already at an advanced stage and is not manageable by radical surgical treatment [4–6]. Despite advances in diagnostic techniques, neoadjuvant chemoradiotherapy and surgery, the treatment outcome of gastric cancer remain poor [7]. For this reason, there is a need to develop innovative, effective therapies.

Angiogenesis is a fundamental stage in the growth of cancers and their metastasis [8–10]. Therefore, the inhibition of angiogenesis has received considerable attention in oncological research, including gastric cancer [8–13]. Anti-angiogenesis treatment inhibits the capillary growth, causing a state of cancer dormancy. This mechanism of anti-angiogenesis therapy was confirmed after the discovery of VEGF family members for the development of stimulators of new blood vessel formation (VEGF A, B, C, D, E) and the discovery of several VEGF pathway targeting drugs [14, 15]. VEGF is an angiogenic factor produced by tumor cells that stimulate the growth of endothelial cells [16]. It enhances the permeability of blood vessels, reduces endothelial cell apoptosis, activates stromal proteolysis, and promotes the proliferation and migration of endothelial cells [17]. VEGF families bind to vascular endothelial growth factor receptors (e.g., VEGFR-1, VEGFR-2, and VEGFR-3). These are all TK receptors that present in both lymphatic and blood vessels endothelial. The binding of VEGF-A to VEGF receptor-2 triggers the dimerisation and transphosphorylation of the intracellular tyrosine kinase domains, leading in activation of TK enzymes and pathways causing cellular proliferation and endothelial cell survival. Inhibition of these pathways by specific inhibitors may result in inhibition of cell proliferation and endothelial cell survival [18]. Clinical treatment techniques were targeting VEGF pathways comprised of an anti-VEGF monoclonal antibody, tyrosine kinase inhibitors and VEGF receptor monoclonal antibodies [18]. Endostatin is a drug that blocks VEGF-induced migration; hence, preventing metastasis of GC through angiogenesis. This drug was developed in China [19].

Many clinical trials have confirmed VEGF inhibitors as important therapeutic agents in multiple solid tumors including gastric cancer. This review aims to assess the treatment outcome of anti-angiogenesis therapy through *VEGF* pathways in the management of gastric cancer patients.

## Main text

### Methods

#### Search strategy

Articles of these clinical trials were identified through a literature search of Pub-Med, MEDLINE and EMBASE. The searches were limited to papers published in the English language. The following strategies were used to retrieve articles, (gastric* OR stomach) AND (cancer OR tumor OR carcinoma) AND (anti-angiogenesis) AND (VEGF) OR (vascular endothelial growth factor) AND (outcome). The literature search started on January 10, 2016, until September 22, 2016. The search was performed independently by reviewers BM and PLC.

#### Study inclusion and exclusion criteria

Eligibility criteria for inclusion in this review were (i) histopathologically diagnosed gastric cancer, (ii) prospective phase II–III trials, (iii) involving adults patients aged 18 years and older, (iv) studies assessing the effect of anti-VEGF agents on patient’s outcomes in the management of advanced gastric cancer, (v) studies published in English.

Exclusion criteria were: (i) non-clinical trials, (ii) non-English literature, (iii) trials with inadequate details of methodologies and results.

#### Data extraction and quality assessment

Eligible studies reviewed and retrieved information was; the name of first author, year of publication, number of patients enrolled, treatment scheme in both arms and outcome measures were (ORR, PFS, OS) and related adverse effects. Consensus resolved any disagreement. Quality of methodology used in each study was assessed independently by reviewers BM and PLC using Cochrane Reviewer’s criteria.

#### Statistical data analysis

In all clinical trials, STATA SE v. 13.1 (STATA_ Corporation, Texas, USA) used for data analysis. Kaplan–Meier method used to calculate median PFS and OS. The HR associated with treatment was estimated using Cox proportional hazards regression. A two-sided alpha of 5% was used to calculate CIs and determine p values. A p value of p < 0.05 was considered significant statistically.

### Results

#### Study selection

**Fig. 1:**
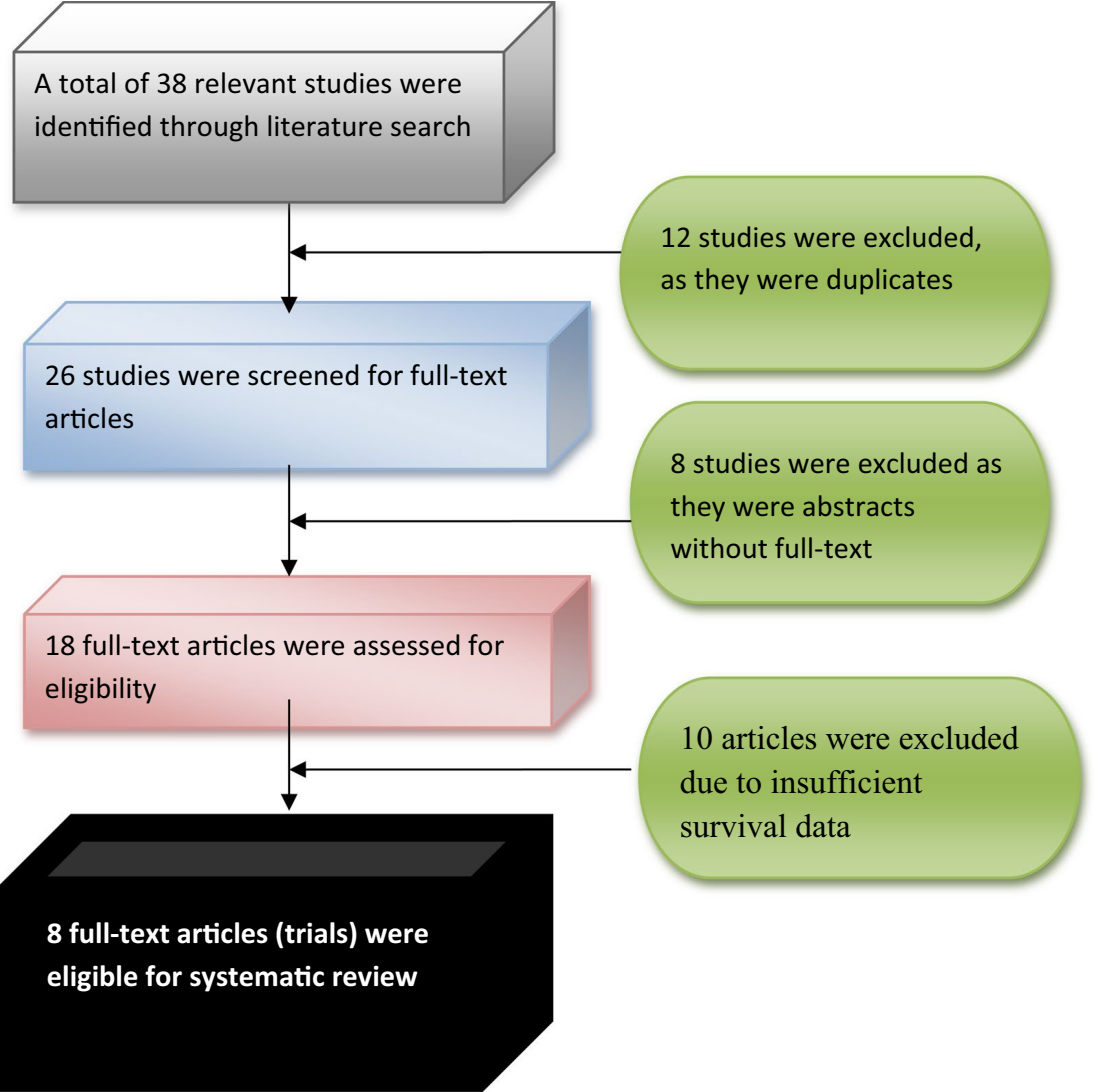
Flow diagram of the studies extracted in this review

38 relevant clinical trials were identified through the literature search. Twelve of studies were excluded due to duplications, leaving 26 full-text articles for further review. 8 studies were excluded because they were not full text. The remaining 18 articles were reassessed, and 10 articles were excluded due to insufficient data. The remaining eight full-text articles were eligible for systematic review (Fig. 1). The title and abstracts of selected articles were reviewed by independent reviewers BM and PLC.

#### Patient/study characteristics

During this review, 2309 patients were included in eight phase II and III clinical trials. The median number of patients in each trial was 288 (range 39–328). In this review, all patients had inoperable, advanced gastric cancer and not manageable by local or regional therapy. Agents under investigation targeted the VEGF pathway including anti-VEGF, the anti-VEGFR monoclonal antibody, and anti-VEGFR tyrosine kinase inhibitors (given either alone or in combination with either chemotherapy or placebo). The outcomes measured reported in all the clinical trials included: overall response rate, progression-free survival, overall survival, and adverse effects.

#### Treatment regimen and outcomes

Anti-VEGF therapies include in phase II, and III trials were:

##### (a) Anti-VEGF monoclonal antibody

Bevacizumab plus chemotherapy has been reported in several clinical trials to increase the anti malignancy activity in some tumors [20–23]. The AVAGAST trial was a phase- III clinical trial that was designed to assess the efficacy of bevacizumab in combination with chemotherapy (capecitabine and cisplatin) in the first-line management of advanced gastric cancer [20]. The results of the AVAGAST trial are shown in Table 1. Therefore in the AVAGAST trial, it was found that adding bevacizumab to chemotherapy could not significantly improve the OS of advanced gastric cancer patients. The AVATAR trial, a phase-III clinical trial that was carried out in China, showed no significant difference between target combined therapy and chemotherapy alone (Table 1). Adverse effects of (Grade C3, C4) for bevacizumab in both arms were similar [24, 25].

**Table 1:** Phase 111 clinical trials of anti-VEGF monoclonal antibody agents in advanced gastric cancer

Variable	AVAGAST trial	AVATAR trial	REGARD trial	RAINBOW trial
Authors	Ohtsu et al.	Shen et al.	Fuchs et al.	Wilke et al.
Number of patients	774	202	355	665
1st-arm	387	100	238	330
2nd-arm	387	102	117	335
Setting	1st-line	1st-line	2nd-line	2nd-line
Treatment regimens				
1st-arm	CT + Bev	CT + Bev	Ram + BSC	PTX + Ram
2nd-arm	CT + placebo	CT + placebo	BSC	PTX + placebo
ORR (%)				
1st-arm (n/%)	46%	41%	3%	28%
2nd-arm	34.4%	34%	3%	16%
HR (95% CI)	8.1 (0.6–16.6)	7.02 (8.3–22.4)		
p value	0.0315	0.34		
TTP/PFS (in months)				
1st-arm	6.7	6.3	2.1	4.40
2nd-arm	5.3	6.0	1.3	2.86
HR (95% CI)	0.80 (0.68–0.93)	0.89 (0.66–1.21)	0.483 (0.376–0.620)	0.635 (0.536–0.752
p value	0.0037	0.47	0.0001	0.0001
OS (in months)				
1st-arm	12.1	10.5	5.2	9.63
2nd-arm	10.1	11.4	3.8	7.26
HR (95% CI)	0.87 (0.73–1.03)	1.11 (0.79–156)	0.77 (0.603–0.998	0.807 (0.678–0.962)
p value	0.1002	0.56	0.047	0.0169

*ORR* median overall response rate, *TTP* median time to progression, *PFS* median progression-free survival, *OS* median overall survival, *T* capecitabine, *C* cisplatin, *Bev* bevacizumab, *HR* hazard ratio, *CI* confidence interval, *Ram* ramucirumab, *BSC* best supportive care, *PTX* paclitaxel

Ramucirumab is a human monoclonal antibody that binds to VEGFR-2 to block the pathways of VEGF in angiogenesis [26]. The REGARD clinical trial was a phase-III trial that was designed to assess the efficacy and safety of ramucirumab in the management of advanced gastric cancer [27]. The patients in the REGARD trial were randomly assigned to receive the best supportive care plus either ramucirumab or a placebo. The analysis of the outcome of this clinical trial demonstrated the improvements in PFS and OS with no sign of improvement of ORR (Table 1).

The RAINBOW trial is another phase-III trial that was designed to evaluate the efficacy of ramucirumab plus chemotherapy (paclitaxel) in one arm and placebo plus chemotherapy (paclitaxel) on the other arm in patients with advanced gastric cancer or gastro-oesophageal junction adenocarcinomas [28]. The results of the RAINBOW trial are summarised in Table 1.

##### (b) Anti-VEGF tyrosine kinase inhibitors (TKIs)

Tyrosine kinase inhibitors are a class of drugs that inhibit VEGF receptors in the management of gastric cancers. These include sunitinib, sorafenib, axitinib, apatinib mesylate, and regorafenib [29]. Sunitinib is a drug that inhibits all members of the VEGFR family. Sunitinib has been investigated as a monotherapy or in combination with chemotherapy in phase-II trials. The results of sunitinib as the monotherapy were as follows: 2 patients had partial responses, and 25 patients had stable disease for ≥ 6 weeks [30]. The results of Sunitinib in combination with chemotherapy were as follows. There was no statistical difference in time progression between two groups [i.e., sunitinib alone (3.9 months) versus target combined chemotherapy (2.6 months)] [30]. The RR was 41.4% versus 14.3% in target combined chemotherapy and sunitinib respectively. Adverse events reported were grade 3, thrombocytopenia in 34.6% and neutropenia in 29.4% of patients. Phase-II clinical trial was designed to evaluate the efficacy and safety of sorafenib in combination with chemotherapy (docetaxel, and cisplatin) in the management of patients with metastatic gastric cancer or gastro-oesophageal adenocarcinomas, in this trial result showed that sorafenib plus chemotherapy was safe but not effective compared to chemotherapy alone [31, 32] (Table 2).

**Table 2:** Phase 11/III clinical trials of anti-VEGF tyrosine kinase inhibitors agents in advanced gastric cancer

Drug	Number of patients	Line	ORR (%)	PFS (months) [HR (95% CI), p value]		OS (months/days) [HR (95% CI), p value]	
Sunitinib	78	2	3.9	31.28		5.81	
Sorafenib	44	1	41	5.8		13.6	
Apatinib (850 mg)	270	1					
Apatinib group	180		2.84	78	HR 0.44, 95% CI 0.33–0.61 p < 0.0001	195 days	HR 0.71, 95% CI 0.54–0.94 p < 0.06
Placebo group	90		0.00	53		140 days	
Trebananib	171	1					
Treb (10 mg)			27	4.2	HR 0.98 95% CI 0.67–1.43 p = 0.92	NR	
Treb (3 mg)			43	4.9		NR	
Ch + placebo			35	5.2		NR	
Regorafenib	147	1					
Rego group	97		NR	11.1		25 weeks	
Placebo group	50		NR	3.9	p = 0.0001	19.4 weeks	p = 0.11

*ORR* median overall response rate, *TTP* median time to progression, *PFS* median progression-free survival, *OS* median overall survival, *HR* hazard ratio, *CI* confidence interval, *Ch* chemotherapy, *Treb* Trebananib, *NR* not reported

Telatinib is an inhibitor of VEGFR-2 and VEGFR-3, in phase-II clinical trial telatinib plus chemotherapy has shown a good toxicity profile in the treatment of gastric carcinomas [33]. In phase-1 clinical trial of apatinib, a TKI blocking VEGFR-2 was evaluated and showed anticancer activities among Chinese patients with metastatic gastric cancer [34–36]. In phase-II trial, 144 patients were involved and assigned to placebo, apatinib 850 mg and apatinib 425 mg. In this trial, apatinib was recommended in the management of gastric cancer. A promising result of apatinib was found in phase-III clinical trial involving 267 patients assigned to two groups (apatinib group and placebo group). The results of this trial are shown in Table 2 [37].

Regorafenib drug was tested in phase-II clinical trial in patients with gastric cancer after they received 1st and 2nd line therapy [38]. Table 2 summarises the results of Regorafenib.

### Discussion

In this systematic review, 2309 patients were involved in eight clinical trials (phase II and III) to assess the treatment outcome of ant-angiogenesis therapy through VEGF in the management of advanced inoperable GC. The results of AVAGAST and AVATAR trials regarding PFS, OS, RR, and toxicities indicate that the effectiveness of bevacizumab in the management of gastric cancer didn’t achieve positive results [20, 39]. RAINBOW and REGARD clinical trials demonstrated that ramucirumab was given alone or in combination with chemotherapy significantly reduced the risk of disease progression (37–52%) and death (19–22%) of gastric cancer [27, 28]. Results of these trials made ramucirumab an effective anti-VEGF drug for local advanced gastric cancer or metastatic gastric cancer.

Bevacizumab is a drug that inhibits VEGF-A only, while ramucirumab inhibits all VEGFs thus inhibiting receptors activation of downstream VEGF signaling pathways resulting in reduced tumor neovascularisation and growth [27]. In the REGARD trial, ramucirumab demonstrated impressive results; hence the Food and Drug Administration approved ramucirumab in the management of advanced and metastatic gastric cancer [27]. Sorafenib, sunitinib and apatinib are TKIs that act by targeting VEGFR pathway. In phase III clinical trial, apatinib (TKI) has shown to improve PFS, and there is a marginal increase in overall survival in patients with chemorefractory GC [37]. A study involving sunitinib as a second-line treatment for advanced gastric cancer results showed insufficient clinical values, while a similar study of sunitinib with conventional chemotherapy revealed severe toxicities [30, 34]. A phase II studies involved sorafenib in combination with chemotherapies in the management of patients with advanced and metastatic gastric cancer, results showed an encouraging efficacy profile with low toxicity, while other study showed very low efficacy profile and the drug was intolerable [30, 31, 38]. Most phase-II randomised clinical trials involving TKI drugs plus chemotherapy present with controversial results [35]. In the Apatinib study, PFS and OS were shown to improve in patients who previously have received two or more chemotherapy regime. Therefore, apatinib remains a standard treatment in patients with disease recurrence after chemotherapy in patients with advanced and metastatic gastric cancer [40].

## Conclusions

This systematic review demonstrated that target combined chemotherapy in the management of advanced gastric cancer showed a significant improvement in OS and ORR over chemotherapy alone. Chemotherapy in combination with anti-VEGFR drugs (e.g., ramucirumab and apatinib) in the management of advanced gastric cancer significantly improves outcome in terms of ORR, PFS, and OS. We recommend that anti-VEGFR pathway drugs be used in the management of patients with advanced gastric cancer.

## Limitations of the study

1. The inclusion of only prospective phase II–III clinical trials published in English may have excluded some informative studies of other phases or published in a non-English language.
2. A high heterogeneity level between a limited number of studies and multiple drugs is involved.

## Abbreviations

CT: chemotherapy
CI: confidence interval
GC: gastric cancer
GEC: gastro-esophageal carcinoma
TKIs: tyrosine kinase inhibitors
HR: hazard ratio
OR: odds ratio
ORR: overall response rate
OS: overall survival
PFS: progression-free survival
RCTs: randomized clinical trials
TK: tyrosine kinase
VEGFR: vascular endothelial growth factor receptor
VEGF: vascular endothelial growth factor
EMBASE: excerpta medical database
MEDLINE: Medical Literature Analysis and Retrieval System Online
AVAGAST: Avastin in gastric cancer trial

## References

[1] Ferlay J, Shin HR, Bray F, Forman D, Mathers C, Parkin DM (2010). Estimates of worldwide burden of cancer in 2008: GLOBOCAN 2008. Int J Cancer.

[2] Torre LA, Bray F, Siegel RL, Ferlay J, Lortet-Tieulent J, Jemal A (2015). Global cancer statistics, 2012. CA Cancer J Clin.

[3] Cunningham D, Allum WH, Stenning SP (2006). Perioperative chemotherapy versus surgery alone for resectable gastroesophageal cancer. N Engl J Med.

[4] Bang YJ, Kim YW, Yang HK (2012). Adjuvant capecitabine and oxaliplatin for gastric cancer after D2 gastrectomy (CLASSIC): a phase 3 open-label, randomized controlled trial. Lancet.

[5] Sasako M, Sakuramoto S, Katai H (2011). Five-year outcomes of a randomized phase III trial comparing adjuvant chemotherapy with S-1 versus surgery alone in stage II or III gastric cancer. J Clin Oncol.

[6] Paoletti X, Oba K, Burzykowski T (2010). Benefit of adjuvant chemotherapy for resectable gastric cancer: a meta-analysis. JAMA.

[7] Lordick F, Allum W, Carneiro F, Mitry E, Tabernero J, Tan P, Van Cutsem E, van de Velde C, Cervantes A (2014). Unmet needs and challenges in gastric cancer: the way forward. Cancer Treat Rev.

[8] Shan F, Miao R, Xue K, Li Z, Li Z, Bu Z, Wu A, Zhang L, Wu X, Zong X, Wang X, Li S, Ji X, Li Z, Ji J (2015). Controlling angiogenesis in gastric cancer: a systematic review of anti-angiogenic trials. Cancer Lett.

[9] Folkman J (1974). Proceedings: tumor angiogenesis factor. Cancer Res.

[10] Folkman J, Watson K, Ingber D, Hanahan D (1989). Induction of angiogenesis during the transition from hyperplasia to neoplasia. Nature.

[11] Kerbel RS (2008). Tumor angiogenesis. N Engl J Med.

[12] Ferrara N, Kerbel RS (2005). Angiogenesis as a therapeutic target. Nature..

[13] Kaspar S, Schuler M (2014). Targeted therapies in gastroesophageal cancer. Eur J Cancer.

[14] Song H, Zhu J, Lu D. Molecular-targeted therapy for advanced gastric cancer. Cochrane Datab Syst Rev. 2015; 1. Art. No.: CD011461. 10.1002/14651858.cd011461. 10.1002/14651858.CD011461.pub2 PMC6457914 27432490

[15] Grothey A, Galanis E (2009). Targeting angiogenesis: progress with anti-VEGF treatment with large molecules. Nat Rev Clin Oncol.

[16] Ferrara N, Gerber HP, LeCouter J (2003). The biology of VEGF and its receptors. Nat Med.

[17] Grothey A, Ellis LM (2008). Targeting angiogenesis driven by vascular endothelial growth factors using antibody-based therapies. Cancer J.

[18] Hicklin DJ, Ellis LM (2005). Role of the vascular endothelial growth factor pathway in tumour growth and angiogenesis. J Clin Oncol.

[19] Park DJ, Thomas NJ, Yoon C, Yoon SS (2015). Vascular endothelial growth factor inhibition in gastric cancer. Gastric Cancer.

[20] Bang YJ, Kang YK, Kang WK (2011). Phase II study of sunitinib as a second-line treatment for advanced gastric cancer. Investig New Drugs.

[21] Shah MA, Jhawer M, Ilson DH, Lefkowitz RA, Robinson E, Capanu M, Kelsen DP (2011). Phase II study of modified docetaxel, cisplatin, and fluorouracil with bevacizumab in patients with metastatic gastroesophageal adenocarcinoma. J Clin Oncol.

[22] El-Rayes BF, Zalupski M, Bekai-Saab T, Heilbrun LK, Hammad N, Patel B, Urba S, Shields AF, Vaishampayan U, Dawson S, Almhanna K, Smith D, Philip PA (2010). A phase II study of bevacizumab, oxaliplatin, and docetaxel in locally advanced and metastatic gastric and gastroesophageal junction cancers. Ann Oncol.

[23] Shields AF, Vaishampayan U, Dawson S, Almhanna K, Smith D, Philip PA (2010). A phase II study of bevacizumab, oxaliplatin, and docetaxel in locally advanced and metastatic gastric and gastroesophageal junction cancers. Ann Oncol.

[24] Shen L, Li J, Xu J, Pan H, Dai G, Qin S, Wang L, Wang J, Yang Z, Shu Y, Xu R, Chen L, Liu Y, Yu S, Bu L, Piao Y (2015). Bevacizumab plus capecitabine and cisplatin in Chinese patients with inoperable locally advanced or metastatic gastric or gastroesophageal junction cancer: randomized, double-blind, phase III study (AVATAR study). Gastric Cancer.

[25] Shen L, Jin L, Xu JM, Pan HM, Dai G, Qin S, Wang L, Wang J, Yang Z, Yongqian S (2012). Efficacy with locally advanced or metastatic gastric/gastroesophageal junction cancer (AGC): results from the AVATAR study. J Clin Oncol.

[26] Wadhwa R, Taketa T, Sudo K, Blum-Murphy M, Ajani JA (2013). Ramucirumab: a novel antiangiogenic agent. Future Oncol.

[27] Fuchs CS, Tomasek J, Yong CJ, Dumitru F, Passalacqua R, Goswami C, Safran H, dos Santos LV, Aprile G, Ferry DR, Melichar B, Tehfe M, Topuzov E, Zalcberg JR, Chau I, Campbell W, Sivanandan C, Pikiel J, Koshiji M, Hsu Y, Liepa AM, Gao L, Schwartz JD, Tabernero J (2014). Ramucirumabmonotherapy for previously treated advanced gastric or gastro-oesophageal junction adenocarcinoma (REGARD): an international, randomized, multicentre, placebo-controlled, phase 3 trial. Lancet.

[28] Wilke H, Muro K, Van Cutsem E (2014). Ramucirumab plus paclitaxel versus placebo plus paclitaxel in patients with previously treated advanced gastric or gastro-oesophageal junction adenocarcinomas (RAINBOW): a double-blind, randomized phase III trial. Lancet Oncol.

[29] Ciliberto D, Staropoli N, Caglioti F, Gualtieri S, Fiorillo L, Chiellino S, De Angelis AM, Mendicino F, Botta C, Caraglia M, Tassone P, Tagliaferri P (2015). A systematic review and meta-analysis of randomized trials on the role of targeted therapy in the management of advanced gastric cancer: evidence does not translate?. Cancer Biol Ther.

[30] Shah MA, Ramanathan RK, Ilson DH, Levnor A, D’Adamo D, O’Reilly E, Tse A, Trocola R, Schwartz L, Capanu M, Schwartz GK, Kelsen DP (2006). Multicenter phase II study of irinotecan, cisplatin, and bevacizumab in patients with metastatic gastric or gastroesophageal junction adenocarcinoma. J Clin Oncol.

[31] Sun W, Powell M, O’Dwyer PJ, Catalano P, Ansari RH, Benson AB (2010). Phase II study of sorafenib in combination with docetaxel and cisplatin in the treatment of metastatic or advanced gastric and gastroesophageal junction adenocarcinoma: ECOG 5203. J Clin Oncol.

[32] Gallego R, Martin-Richard M, Pericay C, Garcia-Foncillas J, Queralt B, Casado E, Feliu J, Vega I, Juez I, Visa L (2012). Phase II study of oxaliplatin and sorafenib in advanced gastric cancer after failure of cis-fluoropyrimidine-based (PF) treatment. J Clin Oncol.

[33] TEL0805 trial. A phase 2 study of telatinib (TEL) in combination with capecitabine (X) and cis (P) as first-line treatment in patients (pts) with advanced gastric or gastro-oesophageal junction (GEJ) cancer. A study of telatinib in combination with chemotherapy in subjects with advanced gastric cancer. http://clinicaltrials.gov/show/NCT00952497/. Accessed 20 Nov 2012.

[34] Li J, Zhao X, Chen L, Guo H, Lv F, Jia K, Yv K, Wang F, Li C, Qian J, Zheng C, Zuo Y (2010). Safety and pharmacokinetics of novel selective vascular endothelial growth factor receptor-2 inhibitor YN968D1 in patients with advanced malignancies. BMC Cancer.

[35] Li J, Qin S, Xu J (2013). Apatinib for chemotherapy-refractory advanced metastatic gastric cancer: results from a randomized, placebo-controlled, parallel-arm, phase IItrial. J Clin Oncol.

[36] Tian S, Quan H, Xie C, Guo H, Lü F, Xu Y, Li J, Lou L (2011). YN968D1 is a novel and selective inhibitor of vascular endothelial growth factor receptor-2 tyrosine kinase with potent activity in vitro and in vivo. Cancer Sci.

[37] Qin S. Phase III study of apatinib in advanced gastric cancer: A randomized, double-blind, placebo-controlled trial. 2014 ASCO Annual Meeting. J Clin Oncol. 2014;32(15)(Suppl):5S. Suppl; abstr 4003.

[38] Wilhelm SM, Carter C, Tang L, Wilkie D, McNabola A, Rong H, Chen C, Zhang X, Vincent P, McHugh M, Cao Y, Shujath J, Gawlak S, Eveleigh D, Rowley B, Liu L, Adnane L, Lynch M, Auclair D, Taylor I, Gedrich R, Voznesensky A, Riedl B, Post LE, Bollag G, Trail PA (2004). BAY 43-9006 exhibits broad spectrum oral antitumor activity and targets the RAF/MEK/ERK pathway and receptor tyrosine kinases involved in tumour progression and angiogenesis. Cancer Res.

[39] Ohtsu A, Shah MA, Van Cutsem E (2011). Bevacizumab in combination with chemotherapy as first-line therapy in advanced gastric cancer: a randomized, double-blind, placebo-controlled phase III study. J Clin Oncol.

[40] Li J, Qin S, Xu J, Guo WJ, Xiong JP, Bai Y, Sun G, Yang Y, Wang L, Xu N, Cheng Y, Zhe-Hai W, Zheng L, Tao M (2011). A randomized, double-blind, multicenter, phase II, three-arm, placebo control study of apatinib as third-line treatment in patients with metastatic gastric carcinoma. J Clin Oncol.

